# Wearable 1 V operating thin-film transistors with solution-processed metal-oxide semiconductor and dielectric films fabricated by deep ultra-violet photo annealing at low temperature

**DOI:** 10.1038/s41598-019-44948-z

**Published:** 2019-06-10

**Authors:** Byoung-Soo Yu, Jun-Young Jeon, Byeong-Cheol Kang, Woobin Lee, Yong-Hoon Kim, Tae-Jun Ha

**Affiliations:** 1 Department of Electronic Materials Engineering, Kwangwoon University, Seoul, 01897 Korea; 2 SKKU Advanced Institute of Nanotechnology (SAINT), Sungkyunkwan University, Suwon, 16419 Korea; 3 School of Advanced Materials Science and Engineering, Sungkyunkwan University, Suwon, 16419 Korea

**Keywords:** Electrical and electronic engineering, Electronic devices

## Abstract

Amorphous metal-oxide semiconductors (AOSs) such as indium-gallium-zinc-oxide (IGZO) as an active channel have attracted substantial interests with regard to high-performance thin-film transistors (TFTs). Recently, intensive and extensive studies of flexible and/or wearable AOS-based TFTs fabricated by solution-process have been reported for emerging approaches based on device configuration and fabrication process. However, several challenges pertaining to practical and effective solution-process technologies remain to be resolved before low-power consuming AOS-based TFTs for wearable electronics can be realized. In this paper, we investigate the non-thermal annealing processes for sol-gel based metal-oxide semiconductor and dielectric films fabricated by deep ultraviolet (DUV) photo and microwave annealing at low temperature, compared to the conventional thermal annealing at high temperature. A comprehensive investigation including a comparative analysis of the effects of DUV photo and microwave annealing on the degree of metal-oxide-metal networks in amorphous IGZO and high-dielectric-constant (high-k) aluminum oxide (Al_2_O_3_) films and device performance of IGZO-TFTs in a comparison with conventional thermal annealing at 400 °C was conducted. We also demonstrate the feasibility of wearable IGZO-TFTs with Al_2_O_3_ dielectrics on solution-processed polyimide films exhibiting a high on/off current ratio of 5 × 10^4^ and field effect mobility up to 1.5 cm^2^/V-s operating at 1 V. In order to reduce the health risk and power consumption during the operation of wearable electronics, the operating voltage of IGZO-TFTs fabricated by non-thermal annealing at low temperature was set below ~1 V. The mechanical stability of wearable IGZO-TFTs fabricated by an all-solution-process except metal electrodes, against cyclic bending tests with diverse radius of curvatures in real-time was investigated. Highly stable and robust flexible IGZO-TFTs without passivation films were achieved even under continuous flexing with a curvature radius of 12 mm.

## Introduction

Over the past few years, amorphous indium-gallium-zinc-oxide (IGZO) has become a promising active channel material for thin-film transistors (TFTs) which have been utilized in different types of electronics, including display backplanes and sensor arrays^1–3^. This is due to its excellent material properties of superb mechanical stability, good electrical conductivity, and optical transparency^4–6^. Compared to silicon-based materials, metal-oxide semiconductors exhibit relatively good electrical characteristics even in an amorphous phase^7–9^. Most high-performance TFTs based on dense inorganic materials have been realized by vacuum processes such as atomic layer deposition and sputtering^10,11^. However, major challenges related to equipment complexity, fabrication costs, and processing capabilities in size and volume can arise with vacuum processes^12,13^. For these reasons, a sol-gel based solution-process as an alternative to the vacuum process has been extensively examined owing to the advantages of simple and practical equipment, low fabrication costs, and large-area deposition capabilities with high throughput^14,15^.

Recently, much effort to reduce the process temperature during the sol-gel based deposition has been made with the upsurge in interest associated with flexible and/or wearable electronics. The conventional annealing process, which requires temperatures as high as 400 °C to decompose the metal precursors used and to reorganize the metal-oxide structures, is not readily applicable to flexible substrates such as paper, rubber, and plastic^16–18^. Hence, several studies focusing on sol-gel based annealing processes at lower temperatures enough to promote the effective condensation and densification of amorphous metal-oxide films have been conducted in an effort to realize high-performance flexible and/or wearable devices^19–21^. Among them, deep-ultraviolet (DUV), and microwave irradiation processes conducted at low temperature enable the formation of high-quality metal-oxide dielectric and semiconducting films by means of photochemical activation energy and electromagnetic vibration energy, respectively^22–25^. Microwave radiation can provide dense metal-oxide films given its use of rapid and volumetric energy realized by the conversion of vibration energy into thermal activation energy^26^. The extensive photon flux generated by DUV irradiation transfers the photo activated energy into the precursor film, which is converted to a dense film with a high degree of metal-oxide-metal (M-O-M) network^27^. The expectation of the non-thermal annealing is placed on its compatibility with flexible substrate, on which conventional thermal annealing at 400 °C precludes the formation of a metal-oxide framework for the fabrication of wearable IGZO-TFTs owing to the serious physical damage.

In addition to the challenge of solution-process at lower temperatures, minimizing the power consumption of TFTs has attracted much attention as increase of efforts associated with scaling down the size and weight of wearable electronics^28,29^. Hence, organic or inorganic dielectrics possessing high dielectric constant (high-k) with suppressed leakage current density have been the topic of research on wearable electronics operating at low voltages^30,31^. In particular, high-k metal-oxide dielectrics such as aluminum oxide (Al_2_O_3_), hafnium oxide (HfO_2_), and zirconium oxide (ZrO_2_) have been utilized given their operational stability at low voltages imparted by their high dielectric constants (>6), wide band gaps (~9 eV), and low interfacial trap states with semiconducting channel films^32–35^. Accordingly, many attempts to fabricate low-voltage operating IGZO-TFTs based on high-k metal-oxide dielectrics have been reported^36–38^. However, most of them were also based on vacuum processes or solution processes at high temperatures ultimately to realize high-quality dielectric films. It is significant to optimize the annealing process carefully so that it is suitable for high-performance wearable TFTs consisting of sol-gel based high-quality metal-oxide semiconductor and dielectric films.

In this paper, we investigate the optimization of DUV photo and microwave annealing in an effort to realize high-quality amorphous IGZO films and characterize charge transport and device performance of IGZO-TFTs fabricated by means of the non-thermal annealing as compared to conventional thermal annealing at 400 °C. The statistical results of the device key metrics in IGZO-TFTs fabricated in different batches at different times are presented to support the effect of the non-thermal annealing process assessed here. We also investigate the origin of high-quality sol-gel based Al_2_O_3_ dielectric films fabricated by DUV photo and microwave annealing at low temperature, in another comparison with conventional thermal annealing. The morphological and structural characteristics of sol-gel based IGZO and Al_2_O_3_ films fabricated by DUV photo and microwave annealing are verified analytically by atomic force microscopy (AFM), scanning electron microscopy (SEM), X-ray diffraction (XRD), and X-ray photoelectron spectroscopy (XPS) measurements. Next, we investigate the optimal configuration of DUV photo and microwave annealing for high-performance low-voltage operating IGZO-TFTs with Al_2_O_3_ dielectrics films. Notably, the studies on the comparative analysis of non-thermal annealing processes for sol-gel based metal-oxide semiconductor and dielectric films in which each annealing processed at low temperature (below ~200 °C) was matched still remain insufficient in terms of device performance of low-voltage operating IGZO-TFTs. Finally, we demonstrate wearable IGZO-TFTs operating at 1 V on solution-processed polyimide (PI) films through the optimized solution-process based on DUV photo annealing, which exhibited good device performance and mechanical stability in cyclic bending tests with a curvature radius of 12 mm.

We note that several studies on solution processes *via* DUV photo and microwave annealing which attempted avoid thermal annealing at high temperatures, have been reported^39,40^. However, most of them focused on either annealing process (microwave or DUV photo annealing) for either sol-gel based metal-oxide film (metal-oxide semiconductor or metal-oxide dielectric). Furthermore, very few have conducted comprehensive investigations of these non-thermal annealing processes that include a detailed comparative analysis for the optimization of high-quality metal-oxide semiconductor and metal-oxide dielectric films compatible with wearable or flexible TFTs operating at 1 V. To the best of our knowledge, this is the first demonstration for the optimized process combination of DUV photo annealing and microwave annealing to realize high-performance IGZO-TFTs consisting of high-quality IGZO semiconducting film and high-k Al_2_O_3_ dielectric with a sufficiently dense M-O-M bonding structure fabricated at low temperature simultaneously, compared to the conventional thermal annealing at high temperature. We believe that analytical comparison of non-thermal annealing processes and thermal annealing process discussed in this study can provide comprehensive understanding for annealing mechanism of sol-gel based metal-oxide materials.

## Results

### Characterization of IGZO-TFTs with a thermally grown SiO_2_ dielectric film

**Figure 1:**
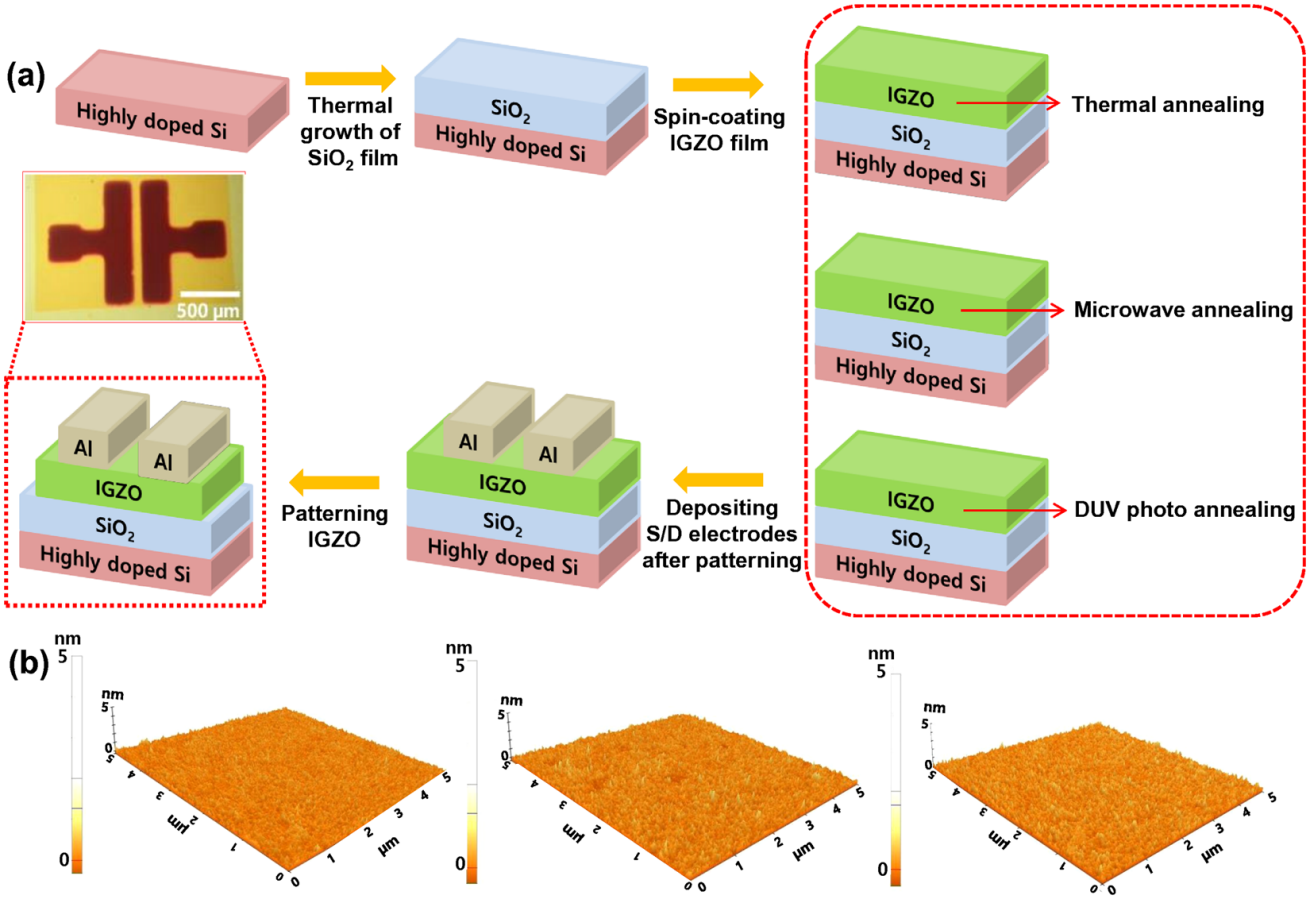
(**a**) Fabrication process flow of IGZO-TFTs with SiO_2_ dielectrics: the inset shows an optical image of the single IGZO-TFT captured by an optical microscope, and (**b**) 3D morphological characteristics of sol-gel based IGZO films fabricated by thermal, microwave, and DUV photo annealing process as obtained from AFM measurements.

The fabrication process flow of solution-processed IGZO-TFTs with conventional SiO_2_ gate dielectrics is schematically shown in Figure 1a with detail explanations in the experimental section. 200 nm thick SiO_2_ dielectric films thermally grown on the silicon substrates were utilized as conventional gate dielectrics. The synthesized IGZO precursor solution was spin-coated on a thermally grown SiO_2_ dielectric/highly doped silicon substrate which was surface-treated with UV-ozone for 20 mins, followed by each fabrication step of thermal annealing, microwave annealing, and DUV photo annealing. The annealing process was crucial to obtain high-quality IGZO films via a sol-gel based deposition in which the activation energy originated from the annealing was directly provided to the gel-type films for the formation of a metal-oxygen-metal (M-O-M) bonding structure^41^. The metal-oxide frameworks were constructed by the condensation of alkoxides/hydroxides via the dissolution of metal precursors in 2-methoxyehanol (2-ME) as a solvent with the removal of residual organic elements including the high concentration of carbon atoms. Furthermore, voids in gel-type films can be significantly densified by the evaporation of the solvents and the decomposition of the precursors through activation annealing^42^. For this reason, the sol-gel based deposition requires thermal annealing at a high process temperature of up to 500 °C to induce the conversion of the metal organic precursors into oxide compounds via the formation of M-O-M networks within the amorphous IGZO films^42^. In order to utilize the sol-gel based deposition on flexible and/or wearable electronics, reducing the process temperature for thermal annealing is essential. However, insufficient activation energy during the annealing process results in poor-quality IGZO films with a high density of defects in the M-O-M network, as well as impurities^43^. As an innovative annealing technology to reduce the process temperature without degrading the quality of the film, DUV photo and microwave annealing processes were performed at low temperature^44^. The maximum process temperatures of the microwave and DUV photo annealing were found to be around 140 °C and 200 °C, respectively, indicating that the temperatures as measured on the surfaces of the films were increased during the annealing process due to the energy absorption. Notably, these temperatures were much lower than those in conventional thermal annealing, which supports the contention that such annealing processes are compatible with flexible and/or wearable substrates such as paper, rubber, and plastic. Charge transport in IGZO-TFTs can be affected by the morphological characteristics of IGZO film as an active channel. Figure 1b shows the morphological characteristics of sol-gel derived IGZO films fabricated by microwave and DUV photo annealing as obtained from AFM measurements. Both IGZO films fabricated by microwave, and DUV photo annealing exhibited significantly clean and smooth surfaces comparable to those fabricated by thermal annealing at 400 °C. The root mean square (RMS) surface roughness of IGZO films annealed by microwave and DUV was similar to the value of thermally annealed IGZO film (Figure S1). These results indicate that microwave and DUV photo annealing resulted in similar morphological characteristics of IGZO films, compared to those by conventional thermal annealing. In order to verify the sol-gel derived IGZO film being semi-conductive, we conducted 4-point probe electrical measurements. The measured resistivity of the IGZO films fabricated by thermal annealing, microwave annealing and DUV photo annealing was 1.5 × 10^−3^, 1.25 × 10^−3^ and 1.03 × 10^−3^ Ω•cm, respectively, which indicates that sol-gel derived IGZO film in this study exhibited semiconducting characteristics.

**Figure 2:**
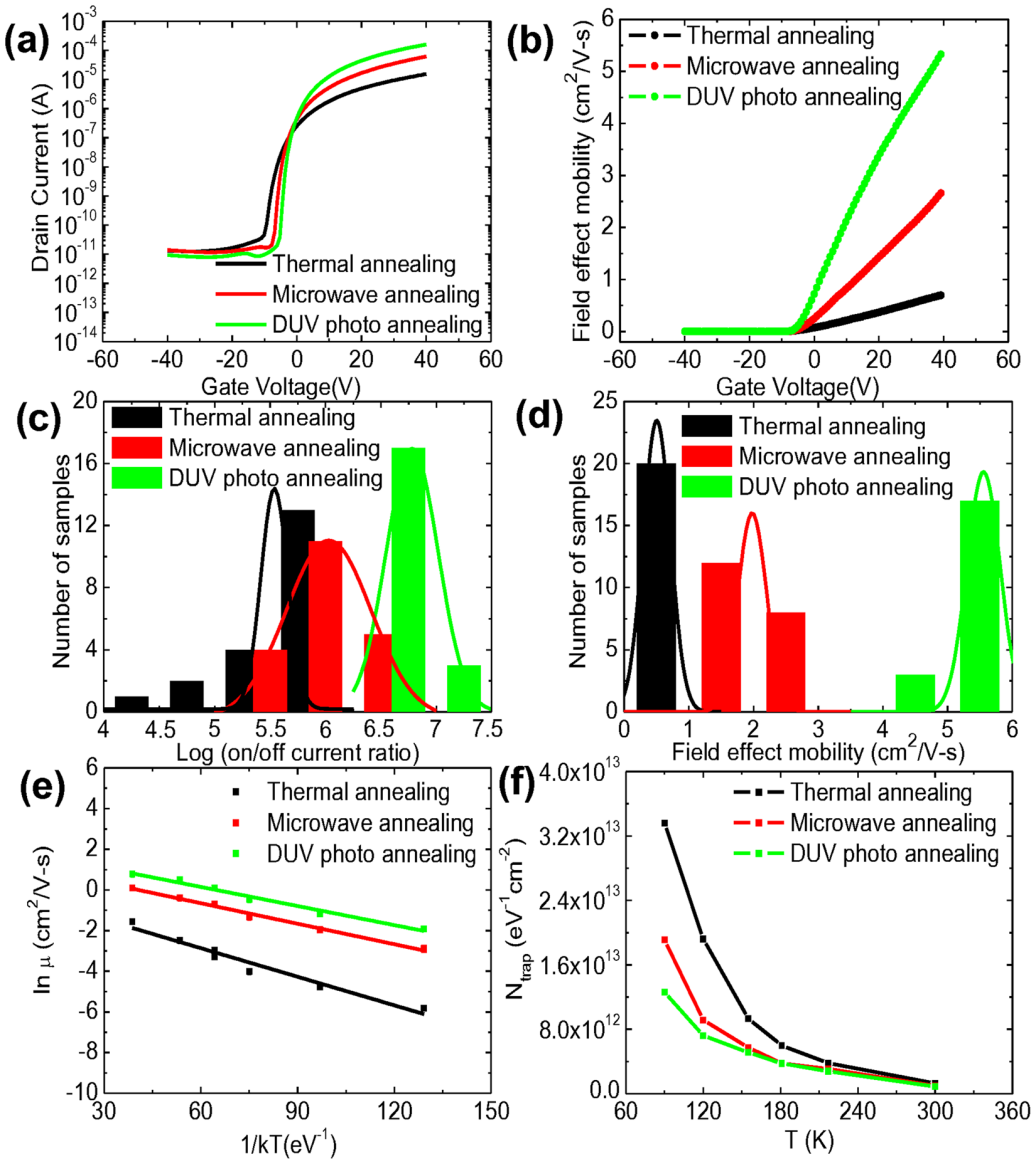
(**a**) Representative transfer curves, and (**b**) field effect mobility of IGZO-TFTs fabricated by thermal, microwave, and DUV photo annealing, and statistical data pertaining to the (**c**) on/off current ratio, and (**d**) field effect mobility of IGZO-TFTs fabricated in different batches at different times, (**e**) a plot of the logarithm of linear field-effect mobilities in IGZO-TFTs fabricated by thermal, microwave, and DUV photo annealing as a function of kT^−1^ and (**f**) the extracted N_trap_ in IGZO-TFTs fabricated by each annealing process as a function of measuring temperature.

Figure 2a,b show the transfer curves and field effect mobility of IGZO-TFTs with SiO_2_ dielectrics fabricated by the thermal, microwave, and DUV photo annealing processes. Conventional SiO_2_ dielectrics which have been generally used for TFTs enable a fair comparison of the effects of each annealing process on the device performance of IGZO-TFTs. With a positive increase in the applied gate voltage, the drain current of the IGZO-TFT increased, providing evidence that electron transport is dominant. As shown in the transfer characteristics, the IGZO-TFTs fabricated by DUV photo annealing process exhibited the highest on/off current ratio of 1.7 × 10^7^, compared to those of 4.44 × 10^6^ and 1.16 × 10^6^ of IGZO-TFTs fabricated by microwave and thermal annealing, respectively. On the basis of the measured drain current (I_ds_), we extracted the field effect mobility (μ) in a linear region at V_ds_ of 5 V using the following equation, which is typically used in the society of semiconductor devices^45^:

1 $${{\rm{I}}}_{{\rm{ds}}}=(\frac{{\rm{W}}}{{\rm{L}}}){{\rm{C}}}_{{\rm{i}}}{\rm{\mu }}[({{\rm{V}}}_{{\rm{gs}}}-{{\rm{V}}}_{{\rm{th}}}){{\rm{V}}}_{{\rm{ds}}}-\frac{{{\rm{V}}}_{{\rm{ds}}}^{2}}{2}]$$

where W, L and C_i_ are the width and length of the channel, and the capacitance of the gate dielectric, respectively. We note that some arguments over overestimation of field-effect mobility extracted from the measured transfer curves have been reported^46,47^. The plot of extracted field-effect mobility as a function of the applied gate voltage shows that the linear field effect mobilities of IGZO-TFTs with SiO_2_ dielectrics fabricated by the thermal, microwave, and DUV photo annealing processes were affected by the gate charge. In TFTs consisting of an active channel film with crystalline structure (or ordered system), band transport is dominant, yielding a field-effect mobility which is constant and independent to gate bias. However, in TFTs consisting of an active channel film with amorphous structure (or disordered system), the major carriers can be easily trapped at localized deep or shallow states, yielding an effective mobility which is affected by the applied gate bias (corresponding to the band bending) Very likely, we observed that the effective mobilities in TFTs with an amorphous metal-oxide semiconducting film were affected by the gate charge. Figure 2b shows that IGZO-TFTs fabricated by DUV photo annealing process exhibited a linear moblity of up to 6 cm^2^/V-s, higher in the same carrier concentration than the others, which indicates that DUV photo annealing is more effective on charge transport in IGZO-TFTs. Figure 2c,d exhibit the statistical data of the on/off current ratio and field-effect mobility in the linear region of IGZO-TFTs fabricated by each annealing process. It is important to note that the 60 samples of IGZO-TFTs used here were fabricated in different batches at different times. The DUV photo annealed IGZO-TFTs exhibited on/off current ratio of 10^7^, whereas the microwave and thermally annealed IGZO-TFTs showed corresponding ratios of ~10^6^ and ~10^5^ on average. The linear mobility of IGZO-TFTs fabricated by DUV photo and microwave annealing processes showed a considerable improvement exceeding factors of 5 and 3, respectively, compared to that by conventional thermal annealing. Furthermore, the DUV photo-annealed IGZO-TFTs exhibited a threshold voltage (V_th_) of −3.31 V and a stiff subthreshold swing (S.S.) as low as 0.55 V/dec, compared to those of IGZO-TFTs fabricated by microwave irradiation (V_th_ of −4.61 V, and S.S. of 0.66 V/dec) and thermal annealing (V_th_ of −6.14 V, and S.S of 0.86 V/dec). In order to investigate the effect of each annealing process on charge transport in IGZO-TFTs, we extracted the activation energy by performing temperature-dependent field-effect mobility measurements in the range from room temperature (~300 K) to 90 K. We note that the electrical characteristics of IGZO-TFTs were re-measured at room temperature after cycling of temperature-dependent measurement and were barely changed compared to the initial. The results indicate that device performances of IGZO-TFTs fabricated by each annealing process were not substantially affected by the temperature cycling^48,49^. Figure 2e shows the Arrhenius plots of IGZO-TFTs fabricated by each annealing process. The activation energy was extracted from the slope of the Arrhenius plots by fitting the equations with the logarithm of the thermally activated mobilities as follows^50^:

2 $${\rm{\mu }}={{\rm{\mu }}}_{{\rm{o}}}{{\rm{e}}}^{-\frac{{{\rm{E}}}_{{\rm{a}}}}{{\rm{kT}}}}$$

where k is the Boltzmann constant, T is a temperature, E_a_ is activation energy, and μ is linear field effect mobility. The activation energy corresponds to the energy distance between the localized trap states and the delocalized band edge, indicating the minimum energy required for contribution in charge transport^51,52^. The extracted activation energy of IGZO-TFTs fabricated by DUV photo annealing was the lowest (31.3 meV), compared to that of IGZO-TFTs fabricated by microwave irradiation (36.3 meV) and thermal annealing (46.9 meV). Lower activation energy of IGZO-TFTs fabricated by DUV photo annealing is in good agreement with better electrical characteristics stemming from the thermally activated charge transport. We also extracted the density of trap states (N_trap_) in the IGZO-TFTs by means of a S.S. analysis via the following equation^53^:

3 $${\rm{S}}.{\rm{S}}.=\frac{{\rm{kTln}}10}{{\rm{e}}}[1+\frac{{{\rm{e}}}^{2}}{{{\rm{C}}}_{{\rm{i}}}}{{\rm{N}}}_{{\rm{trap}}}]$$

where k is the Boltzmann constant, T is the temperature, and C_i_ is the capacitance per unit area. N_trap_ at the interfaces of IGZO-TFTs fabricated by DUV photo annealing was 8.7 × 10^11^ cm^−2^ eV^−1^, which is lower than those of the others (1.06 × 10^12^ cm^−2^ eV^−1^ by microwave annealing, and 1.41 × 10^12^ cm^−2^ eV^−1^ by thermal annealing). Figure 2f shows the extracted N_trap_ in IGZO-TFTs fabricated by each annealing process as a function of measuring temperature. N_trap_ of IGZO-TFTs decreased with increasing the measuring temperature regadless of annealing process, which indicates that low thermal-assistant activation at low temperatures leads to the insufficient release of charge carriers from the trap states^54^. As expected, the DUV photo annealed IGZO-TFTs revealed the lowest N_trap_ in all ranges of temperatures due to lower activation energy^55,56^. Lower density of trap states in sol-gel based IGZO films resulted in better charge transport in the IGZO-TFTs thereby improving the device performance^57^. High performance of IGZO-TFTs fabricated by DUV photo and microwave annealing can be expalined by high-quality IGZO films with improved structural characteristics.

**Figure 3:**
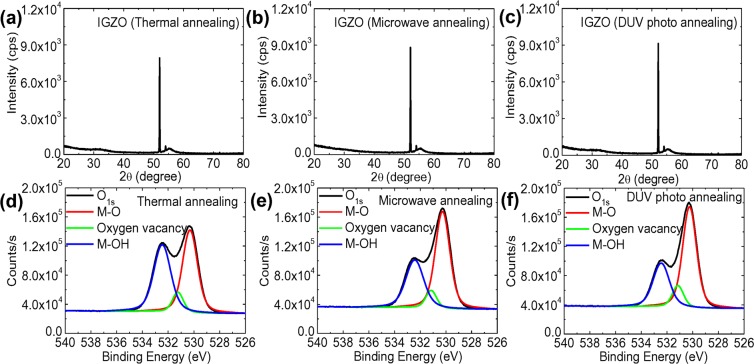
XRD patterns of IGZO films fabricated by (**a**) thermal, (**b**) microwave and (**c**) DUV photo annealing processes, and O_1s_ XPS spectra of IGZO films fabricated by (**d**) thermal, (**e**) microwave and (**f**) DUV photo annealing processes.

XRD patterns of the IGZO films fabricated by three different annealing processes are shown in Figure 3a–c. Only one diffraction peak corresponding to the silicon substrate was observed at approximately 52.1° without any sharp diffraction peaks caused by the crystalline structure. These results indicate that the sol-gel based IGZO films possess an amorphous phase structure regardless of the annealing processes used, supporting the contention that the crystalline structure in the IGZO films was not induced by DUV photo or microwave annealing^58^. Subsequently, we investigated the effects of these annealing processes on the structural bonding states in the IGZO films through XPS measurements. As shown in Figure 3d–f, the O_1s_ spectra of the IGZO films were deconvoluted into three different peaks according to a Gaussian fitting distribution^59,60^. Three binding energy peaks in the O_1s_ spectra were found at 530.3 ± 0.1, 531.2 ± 0.1, and 532.4 ± 0.1 eV^60^. The lowest binding energy peak is defined as an oxygen lattice bond (M-O bonding), indicating that O^2−^ ions combined with three metal atoms in the IGZO films^60^. The oxygen deficiency peak stems from oxygen vacancies caused by O^2−^ ions in oxygen deficient regions^61^. The surface oxygen hydroxide bonds (M-OH bonding) centered at the highest binding energy peak are related to weakly bound oxygen, which can be structurally changed by chemical adsorption with residual components such as the carbon content and/or the hydroxyl group^61^. With regard to the proportional ratios of the M-O bonding states in the sol-gel based IGZO films, the DUV photo-activated and microwave-annealed IGZO films exhibited relatively more M-O bonding states of 59.38 and 57.52%, respectively, compared to the portion of 47.56% observed in the IGZO films fabricated by conventional thermal annealing at 400 °C. Furthermore, non-thermal annealing resulted in a lower ratio of M-OH bonding states, associated with nitrogen ligands and organic molecules in the as-deposited IGZO films. Residual solvent molecules such as carbon and nitrogen components were efficiently decomposed into diffusible molecules by extensive photoradical and vibration-based activation^62,63^. Hence, DUV photo and microwave annealing promoted superb structural integrity of dense M-O-M networks in the sol-gel based IGZO films by inducing a high degree of condensation of the metal alkoxides/hydroxides and densification of the film originating from chemically and structurally stable oxygen with the sufficient removal of chemical impurities^62,63^. This improved quality of IGZO films fabricated by non-thermal annealing at low temperature is in good agreement with improved device performance of IGZO-TFTs, as discussed above.

### Characterization of sol-gel based high-k dielectric Al_2_O_3_ films

**Figure 4:**
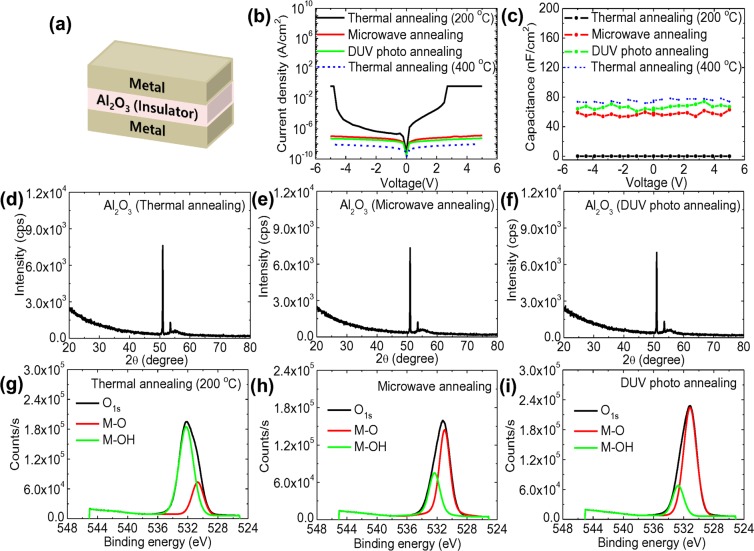
(**a**) Cross section of a MIM device with Al_2_O_3_ dielectric film, and (**b**) J-V and (**c**) C-V characteristics of a MIM structure consisting of sol-gel based Al_2_O_3_ dielectric films fabricated by thermal (200, and 400 °C), microwave and DUV photo annealing processes, XRD patterns of Al_2_O_3_ dielectric films fabricated by (**d**) thermal, (**e**) microwave and (**f**) DUV photo annealing, and O_1s_ XPS spectra of Al_2_O_3_ dielectric films fabricated by (**g**) thermal (200 °C), (**h**) microwave and (**i**) DUV photo annealing processes.

Sol-gel based Al_2_O_3_ dielectric films by thermal annealing at temperature lower than 400 °C exhibited poor dielectric characteristics due to the high density of defect states including pin-holes and impurities^64^. Akin to the sol-gel based IGZO films, we investigated the effect of non-thermal annealing on the dielectric characteristics of Al_2_O_3_ films fabricated by solution-process at low temperature using a metal-insulator-metal (MIM) structure, as shown in Figure 4a. The morphological properties of sol-gel based Al_2_O_3_ films by each annealing process were investigated through AFM measurements (Figure S2a–c). Notably, charge scattering induced by certain morphological characteristics at the interfaces of dielectric films can affect charge transport in IGZO-TFTs^65,66^. Sol-gel based Al_2_O_3_ films exhibited very smooth and flat surface with roughness of ~0.12 nm regardless of annealing process (Figure S2d). In order to investigate the dielectric properties of Al_2_O_3_ films fabricated by DUV photo and microwave annealing, we characterized the leakage current densities of the MIM structure as a function of the applied voltage at 5 V, as exhibited in Figure 4b. The leakage current density at 1 MV/cm in Al_2_O_3_ films fabricated by microwave and DUV photo annealing was significantly suppressed to 1.29 × 10^−7^ A/cm^2^ and 5.60 × 10^−8^ A/cm^2^, respectively, comparable to that by conventional thermal annealing at 400 °C. We observed that the operation of the MIM devices consisting of Al_2_O_3_ dielectric films fabricated by thermal annealing at 200 °C failed by the large leakage current. The severe degradation of the dielectric properties is presumed to result from the numerous organic carbon components and hydroxyl groups, which leads to the formation of leakage current paths due to insufficient thermal activation^67^. The areal capacitance values of the sol-gel based Al_2_O_3_ films by each annealing method at 1 kHz as a function of the applied voltage are shown in Figure 4c. The measured capacitance of Al_2_O_3_ films fabricated by microwave and DUV photo annealing are 62.91 and 74.07 nF/cm^2^, respectively, comparable to that by conventional thermal annealing at 400 °C. We extracted the dielectric constant of Al_2_O_3_ films using a following equation in order to verify the sol-gel derived Al_2_O_3_ film being high-K:

4 $${\rm{C}}={{\rm{\varepsilon }}}_{{\rm{o}}}{{\rm{\varepsilon }}}_{{\rm{r}}}\frac{{\rm{A}}}{{\rm{d}}}$$

where C is the capacitance, ε_o_ is the vacuum permittivity, ε_r_ is the dielectric constant, A is the area of metal electrode and d is the distance between the two sides of the metal. In order to measure the thickness of Al_2_O_3_ film, we conducted scanning electron microscopy (SEM) measurements. As shown in Figure S3, the thicknesses of sol-gel derived Al_2_O_3_ films by thermal annealing at 400 °C, DUV photo annealing and microwave annealing, as obtained from SEM measurements were 82, 82, and 81 nm, respectively. As a result, the extracted dielectric constants of the sol-gel derived Al_2_O_3_ films was ~6. We also investigated XRD patterns of high-k Al_2_O_3_ dielectric films fabricated by three different annealing processes. As shown in Figure 4d–f, XRD patterns of the Al_2_O_3_ films fabricated by three different annealing processes exhibited no sharp diffraction peak related to the crystalline structure. These results clearly indicate that the sol-gel derived Al_2_O_3_ films in this study exhibit an amorphous phase structure regardless of the annealing processes. The enhanced dielectric performance of the MIM devices consisting of Al_2_O_3_ dielectric films fabricated by DUV photo and microwave annealing originated from the high degree of condensation and densification in the films with the enhanced dehydroxylation caused by intense photochemical and vibration-based activation, compared to thermal annealing at 200 °C^68^. Figure 4g–i show the O_1s_ spectra of sol-gel derived Al_2_O_3_ dielectric films by each annealing process where the two peaks located at 530.9 ± 0.2 and 532.5 ± 0.2 eV are applicable to M-O and M-OH bonding, respectively. Sol-gel based Al_2_O_3_ films formed by thermal annealing at 200 °C exhibited considerably high areal ratios of their M-OH bonding states (76.69%) compared to that of the M-O bonding states (23.31%) whereas Al_2_O_3_ films formed by microwave and DUV photo annealing exhibited M-O bonding states of 63.40% and 80.82%, respectively. These results indicate that thermal annealing at 200 °C resulted in the considerable deterioration of the Al_2_O_3_ films due to the incomplete formation of the M-O-M network and the numerous chemical impurities associated with defect states^69^. However, the conversion of large amounts of hydroxyl groups into dense oxygen lattice bonds was induced by DUV photo and microwave annealing, supporting the sufficient decomposition process of the organic components and metal precursors even at a lower temperature^70^.

### Process optimization to fabricate low-voltage operating IGZO-TFTs with Al_2_O_3_ dielectric films by non-thermal annealing at low temperature

**Figure 5:**
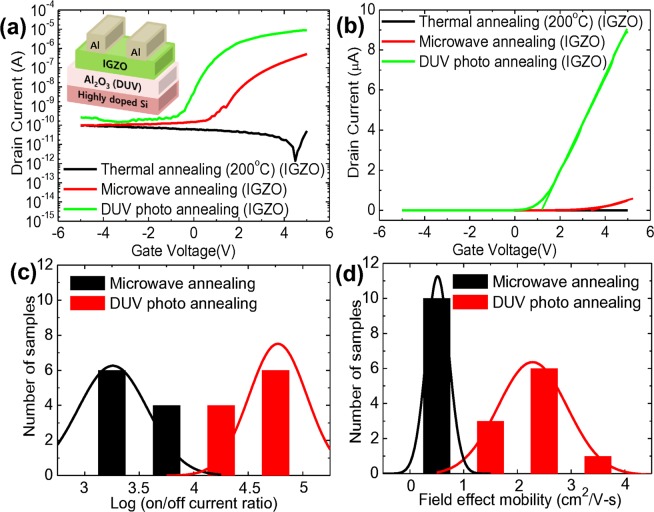
(**a**) Representative transfer characteristics of IGZO-TFTs with Al_2_O_3_ dielectric fabricated by thermal (200 °C), microwave, DUV photo annealing processes at V_DS_ of 1 V: the inset shows a cross-section of an IGZO-TFT with DUV annealed Al_2_O_3_ dielectric film, (**b**) transfer curves on a linear scale of IGZO-TFTs with Al_2_O_3_ dielectrics fabricated by thermal (200 °C), microwave, and DUV photo annealing processes, and statistical data of (**c**) on/off current ratio, and (**d**) field effect mobility of IGZO-TFTs with Al_2_O_3_ dielectrics fabricated in different batches at different times.

In order to realize high-performance solution-processed IGZO-TFTs with Al_2_O_3_ dielectric films operating at low voltages, we investigated the process optimization for DUV photo and microwave annealing. Based on photo-chemically activated Al_2_O_3_ films exhibiting relatively better dielectric characteristics, the device performance of TFTs consisting of sol-gel based IGZO films by DUV photo and microwave annealing processes was characterized, as shown in Figure 5a. As expected, the failure in the operation of IGZO-TFTs fabricated by thermal annealing at 200 °C was observed. TFTs consisting of DUV activated IGZO films exhibited better device performance, showing an on/off current ratio of ~10^5^, a V_th_ of 0.11 V, and a S.S. of 0.38 V/dec than those of microwave-activated IGZO films with an on/off current ratio of ~10^4^, a V_th_ of 2.17 V, and a S.S. of 0.53 V/dec. Figure 5b shows the transfer curves of IGZO-TFTs fabricated by each annealing process on a linear scale, where the slope indicates the field-effect mobility. The field-effect mobility of a TFT with a DUV activated IGZO semiconductor on a DUV activated Al_2_O_3_ dielectric was improved by a factor of 5, compared to that of a TFT with a microwave-activated IGZO semiconductor on such, in good agreement with the outcomes for TFTs on SiO_2_ dielectrics. Figure 5c,d show the statistical data of the on/off current ratio, and field effect mobility of IGZO-TFTs fabricated by microwave and DUV photo annealing processes, as extracted from 20 samples fabricated in different batches at different times. The results conclusively support that high performance IGZO-TFTs operating at low voltages can be realized by a careful process optimization for DUV activated IGZO semiconductor and Al_2_O_3_ dielectric films.

### Low-voltage operating flexible IGZO-TFTs fabricated by DUV photo annealing for wearable electronics

Low power consumption has been investigated intensively with regard to wearable electronics given the increased interest in safety and portability. It is significant to reduce the operating voltages to below 1 V for TFTs in highly effective and practical wearable electronics. The electromagnetic energy generated by microwave absorption was transferred to thermal activation energy through the energy conversion step in which volumetric annealing was directly delivered to the metal-oxide films^71^. This technique can induce structural integration in M-O-M networks within metal-oxide films in a rapid time instead of thermal annealing process at high temperatures. However, microwave radiation may result in damage to organic/polymeric substrates which arise during the deposition process of metal-oxide films. On the other hand, photo energy from the high photon flux generated by DUV irradiation directly contributed to the highly effective condensation and densification of metal-oxide films without negative effects on the substrates^72^. Notably, DUV photo-activated annealing is a promising candidate to realize high-quality metal-oxide films without physical and/or chemical damage to flexible substrates. To develop high-performance wearable IGZO-TFTs operating at 1 V, we undertook the process optimization of DUV photo annealing for IGZO-TFTs based on flexible substrates, PI films in this study.

**Figure 6:**
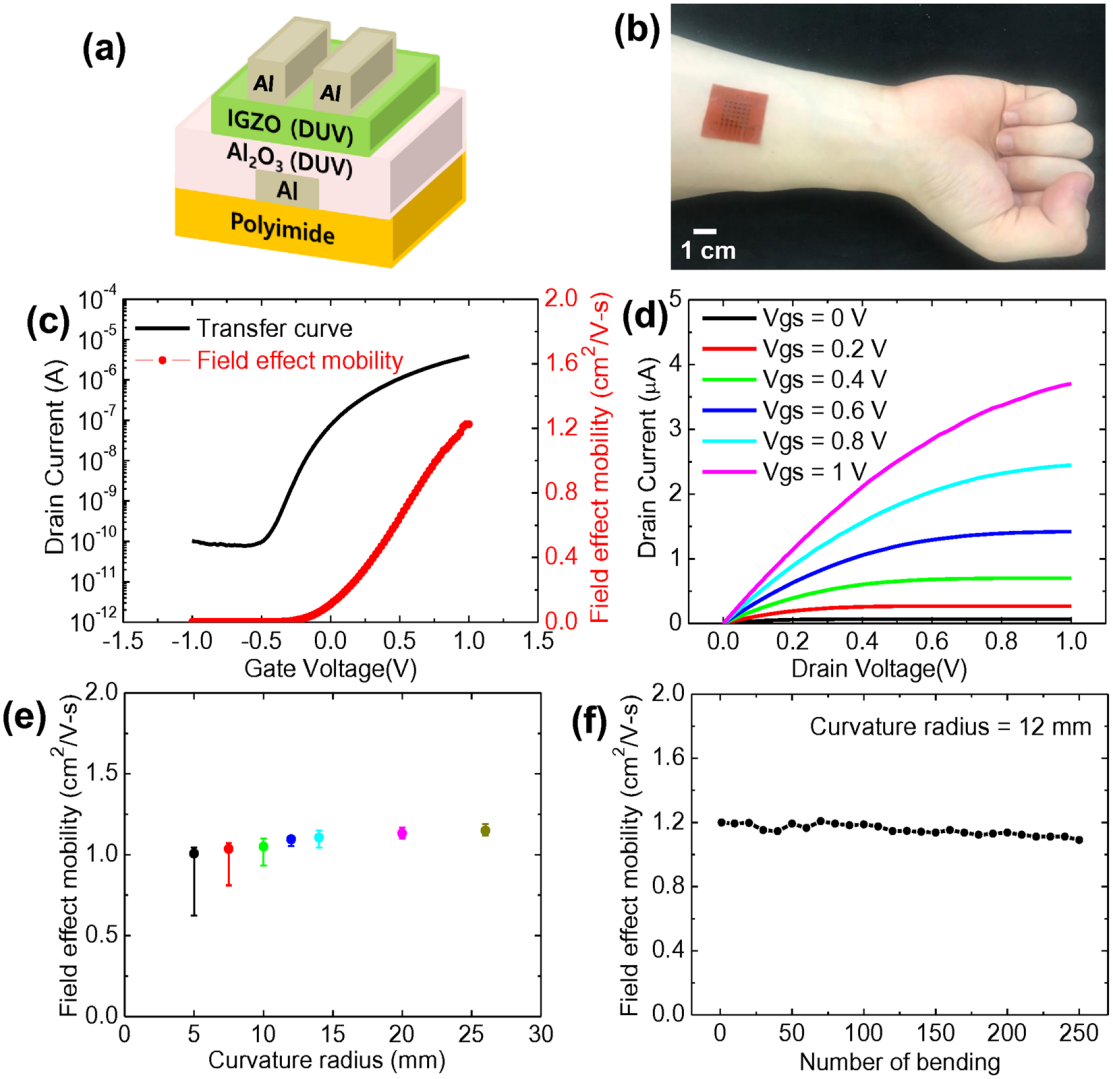
(**a**) Cross-section of solution-processed IGZO-TFTs with Al_2_O_3_ dielectrics fabricated by DUV photo annealing on flexible PI film, (**b**) an optical image of wearable IGZO-TFT array on a PI film directly attached to a human arm, (**c**) a representative transfer curve and field effect mobility at V_DS_ of 0.2 V and (**d**) output curves of flexible IGZO-TFTs, and field effect mobility of flexible IGZO-TFTs as a function of (**e**) the curvature radius (mm), and (**f**) the number of bending for a curvature radius of 12 mm.

A schematic cross-section and the detailed fabrication process of the solution-processed IGZO-TFTs on PI films are shown in Figures 6a and S4, respectively. Very thin PI film can be attached to the human skin after careful releasing it from the glass substrate thereby maintaining the device performance of solution-processed IGZO-TFTs without physical damage caused by external stress. In order to realize 1 V operating IGZO-TFTs, we optimized the capacitance of sol-gel derived Al_2_O_3_ dielectric film with different molar concentrations (0.8 M, 0.4 M, 0.2 M and 0.15 M) by DUV photo annealing. As shown in Figure S5, the thickness of DUV photo annealed Al_2_O_3_ dielectric film was decreased from 83 to 19 nm with the molar concentrations. Accordingly, the measured capacitance of sol-gel derived Al_2_O_3_ dielectric film with a molar concentration of 0.15 M by DUV photo annealing was increased to ~400 nF/cm^2^, as shown in Figure S6. Figure 6b shows an optical image of a wearable IGZO-TFT array on PI film after it was detached from the handling glass substrates and attached to a human arm^73^. The key performance metrics of the devices in the transfer curve, field effect mobility and output curve of the flexible IGZO-TFTs with Al_2_O_3_ dielectrics are described in Figure 6c,d. The optimized flexible IGZO-TFTs operating at 1 V exhibited good transfer and output characteristics with an on/off current ratio of ~5 × 10^4^, a field effect mobility of 1.23 cm^2^/V-s, and a S.S. of 0.12 V/dec. The density of the trap states (N_trap_) of an IGZO-TFT on a PI substrate was extracted and found to be 2.73 × 10^12^ cm^−2^eV^−1^, larger than that of an IGZO-TFT on a silicon substrate. In addition to the good electrical characteristics, the mechanical stability of flexible IGZO-TFTs against applied exterior force is essential for high operational stability and durability of wearable electronics. For this reason, we performed cyclic bending tests of the proposed flexible IGZO-TFTs using a custom-designed strain measurement machine (Figure S7a). Figure 6e shows the field effect mobilities of flexible IGZO-TFTs fabricated by DUV photo annealing as a function of the curvature radius at 5, 7.5, 10, 12, 14, 20, and 26 mm. The field effect mobility was mostly unchanged with the degree of curvature radius up to 12 mm, indicating that flexible IGZO-TFTs exhibited outstanding mechanical robustness. We note that such achievements were realized in flexible IGZO-TFTs without passivation films. However, the reliability against the cyclic bending test under harsh condition with smaller curvature radius of 10, 7.5 and 5 mm was not fully guaranteed. Large variation in the electrical properties of flexible IGZO-TFTs after bending test under harsh condition is presumed to result from the thin PI substrate and non-passivation. The suitable thickness of flexible substrate for wearable IGZO-TFTs is the key to realizing wearability. If the PI flexible substrate is thicker, it is very difficult to attach flexible IGZO-TFTs to the human skin. For this reason, we utilized very thin PI film as a flexible substrate fabricated by solution-process. By implementing a suitable passivation film to control the position of the neutral plane in the metal-oxide films, further improvement in the mechanical stability can be expected^74^. We note that the mechanical stability of wearable IGZO-TFTs at extremely small curvature radiuses is not largely significant for wearability. Figures 6f and S7b show the operational stability of flexible IGZO-TFTs fabricated by DUV photo annealing after repetitive bending cycle of 250 times with a curvature radius of 12 mm. Apart from a slight shift in V_th_ in the transfer curves, serious degradation in the electrical properties of flexible IGZO-TFTs fabricated in this study was not observed, even after consecutive bending stress tests with a curvature radius of 12 mm. This provides evidence that highly stable and robust flexible IGZO-TFTs operating at 1 V were realized by the optimized fabrication process of DUV photo annealing which is suitable for high-performance flexible and/or wearable electronics with low power consumption.

## Discussion

We demonstrated high-performance 1 V operating wearable IGZO-TFTs with high-k Al_2_O_3_ dielectric films by process optimization for non-thermal DUV photo annealing. The characteristics of sol-gel derived IGZO and Al_2_O_3_ films by DUV photo and microwave annealing at low temperature were comparable to those fabricated by conventional thermal annealing at 400 °C. We also investigated the origin of high-quality sol-gel derived metal-oxide semiconductor and dielectric films by microwave and DUV photo annealing regarding M-O bonding states in an effort to investigate the annealing mechanism for device performance of low-voltage operating wearable IGZO-TFTs. Finally, we investigated the operational stability of wearable IGZO-TFTs on PI films by an all-solution-process except metal electrodes involving DUV photo annealing, against cyclic bending tests with diverse radius of curvatures in real-time. Highly stable and robust flexible IGZO-TFTs without passivation films were achieved even under continuous flexing with a curvature radius of 12 mm. We believe that this work provides a new route for high-performance low-voltage operating wearable TFTs based on sol-gel based metal-oxide semiconductor and dielectric films through DUV photo and microwave annealing at low temperature toward flexible and/or wearable electronics with low power consumption.

## Methods

### Synthesis of IGZO and Al_2_O_3_ solution

The IGZO solution (0.125 M) was prepared by dissolving indium nitrate hydrate (In(NO_3_)_3_ • xH_2_O), gallium nitrate hydrate (Ga(NO_3_)_3_ • xH_2_O) and zinc nitrate hydrate (Zn(NO_3_)_2_ • xH_2_O) powders (Sigma Aldrich) in 2-methoxyethanol (2-ME) as a solvent (anhydrous, Sigma Aldrich). The Al_2_O_3_ solution (0.8 M) was also prepared by dissolving aluminum nitrate nonahydrate (Al(NO_3_)_3_ • 9H_2_O) (Sigma Aldrich) powder in 2-ME as a solvent (anhydrous, Sigma Aldrich). The molar concentration of the Al_2_O_3_ solution used to construct flexible IGZO-TFTs was optimized to 0.15 M, which enables the device to operate at 1 V. The IGZO and Al_2_O_3_ solutions were magnetically stirred at 70 °C for 12 h at an ambient atmosphere.

### Fabrication process for IGZO-TFTs

IGZO-TFTs were fabricated on highly doped p-type silicon substrates with resistivity of ~0.01 Ω∙cm for the gate electrodes. For a fair comparison of the device performance in TFTs consisting of IGZO active channels fabricated by different annealing processes, 200 nm thick SiO_2_ dielectric films thermally grown on silicon substrates were utilized as conventional gate dielectrics. The Al_2_O_3_ dielectric films were deposited on to silicon substrates by spin coating at 6000 rpm for 45 s, followed by the different annealing processes. The samples were cleaned with organic solvents of acetone, methanol, and isopropyl alcohol for each 10 minute, followed by treatments with UV ozone in order to make them hydrophilic. The IGZO solution was spin-coated onto the surface-treated SiO_2_ dielectric at 4000 rpm for 45 s, followed by thermal, microwave, or DUV photo annealing process. In order to form high-quality amorphous IGZO films, thermal annealing process at a high temperature of 400 °C was conducted for 1 h. Microwave annealing at a power of 700 W and frequency of 60 Hz was processed for 15 min in an ambient atmosphere and DUV photo annealing with the emission wavelengths of 253.7 nm (90%) and 184.9 nm (10%) (UV253H, Filgen) and the output energy intensity of the lamp was 25 mW/cm2 was performed for 2 h under nitrogen-purging atmosphere, respectively. 75 nm-thick aluminum (Al) source and drain (S/D) electrodes were deposited by thermal evaporation under a vacuum of ~10^6^ torr after patterning. Finally, channel patterning was performed by photolithography and wet-etching to complete the bottom-gate-top-contact IGZO-TFTs.

### Fabrication process for flexible IGZO-TFTs

The detailed fabrication process for flexible IGZO-TFTs operating at 1 V is described in Figure S4. Solution-processed PI films (PicoMAX Co. POLYZEN 150 P) with a thickness of 20 μm were deposited on the surface-treated glass substrate as a handling substrate, followed by the formation of a gate electrode with 30 nm-thick Al film. Al_2_O_3_ and IGZO films were deposited by spin coating, followed by the optimized DUV photo annealing consecutively. As described above, Al S/D electrodes were deposited by thermal evaporation, and this was followed by channel patterning. The IGZO-TFTs fabricated in this work possess a channel width of 1000 µm and a channel length of 50 µm, respectively. An optical image of a single IGZO-TFT was captured by an optical microscope (Figure 1a, inset)

### Device characterization

The leakage current density and areal capacitance as a function of the applied voltage in MIM structures consisting of sol-gel based Al_2_O_3_ films where an area of metal electrode was 0.00225 cm^2^ were characterized using a semiconductor parameter analyzer and a LCR meter, respectively. The electrical properties of transfer and output characteristics in IGZO-TFTs were also characterized by the semiconductor parameter analyzer. Temperature-dependent field-effect mobility measurements were conducted using Vacuum Probe Station with liquid nitrogen under pressure of ~10^−2^ Torr in the range from 300 K to 90 K.

## Supplementary information

supporting information
