# Supplementary material for: Wearable 1 V operating thin-film transistors with solution-processed metal-oxide semiconductor and dielectric films fabricated by deep ultra-violet photo annealing at low temperature
Source: Sci Rep. 2019 Jun 10;9:8416. doi: 10.1038/s41598-019-44948-z (PMC6558031; doi:10.1038/s41598-019-44948-z)
Supplement: Supplementary file 1 — supporting information [file 41598_2019_44948_MOESM1_ESM.docx]

**Wearable 1 V operating thin-film transistors with solution-processed metal-oxide semiconductor and dielectric films fabricated by deep ultra-violet photo annealing at low temperature**

Byoung-Soo Yu^1,†^, Jun-Young Jeon^1,†^, Byeong-Choel Kang^1^, Woobin Lee^2^, Yong-Hoon Kim^2,3,*^ & Tae-Jun Ha^1,*^

^1^Department of Electronic Materials Engineering, Kwangwoon University, Seoul 01897, Korea

^2^SKKU Advanced Institute of Nanotechnology (SAINT), Sungkyunkwan University, Suwon 16419, Korea

^3^School of Advanced Materials Science and Engineering, Sungkyunkwan University, Suwon 16419, Korea

*Corresponding author: [taejunha0604@gmail.com](file:///C:\Users\AMDEL_BSPark\Desktop\전준영%202018\키스트\ACS%20gas%20sensor(최종%20제출본)\taejunha0604@gmail.com), yhkim76@skku.edu

**Figure S1.** Surface roughness of solution-processed IGZO films fabricated by each annealing process.

**Figure S2.** Morphological characteristics of solution-processed Al_2_O_3_ dielectric films fabricated by (a) thermal, (b) microwave, and (c) DUV photo annealing processes, and (d) surface roughness of solution-processed Al_2_O_3_ films fabricated by each annealing process.

**Figure S3.** The thicknesses of sol-gel derived Al_2_O_3_ films by (a) thermal annealing at 400 ^o^C, (b) DUV photo annealing and (c) microwave annealing, as obtained from SEM measurements

**Figure S4.** The fabrication process flow of flexible IGZO-TFTs with Al_2_O_3_ dielectrics.

**Figure S5.** The thickness of sol-gel derived Al_2_O_3_ dielectric film with different molar concentrations of (a) 0.8 M, (b) 0.4 M, (c) 0.2 M and (d) 0.15 M by DUV photo annealing

**Figure S6.** C-V characteristic of a MIM structure consisting of DUV photo annealed Al_2_O_3_ dielectric film with a molar concentration of 0.15 M

**Figure S7. (**a) Custom-designed strain measurement machine used for the cyclic bending tests, and (b) transfer curves of flexible IGZO-TFTs after repetitive bending cycle of 250 times with a curvature radius of 12 mm
